# Monitoring and Evaluating the Transition of Large-Scale Programs in Global Health

**DOI:** 10.9745/GHSP-D-15-00221

**Published:** 2015-12-15

**Authors:** James Bao, Daniela C Rodriguez, Ligia Paina, Sachiko Ozawa, Sara Bennett

**Affiliations:** ^a^​ University of Toronto, Faculty of Medicine, Toronto, Ontario, Canada; ^b^​ Johns Hopkins Bloomberg School of Public Health, Department of International Health, Baltimore, MD, USA

## Abstract

**Purpose:**

Donors are increasingly interested in the transition and sustainability of global health programs as priorities shift and external funding declines. Systematic and high-quality monitoring and evaluation (M&E) of such processes is rare. We propose a framework and related guiding questions to systematize the M&E of global health program transitions.

**Methods:**

We conducted stakeholder interviews, searched the peer-reviewed and gray literature, gathered feedback from key informants, and reflected on author experiences to build a framework on M&E of transition and to develop guiding questions.

**Findings:**

The conceptual framework models transition as a process spanning pre-transition and transition itself and extending into sustained services and outcomes. Key transition domains include leadership, financing, programming, and service delivery, and relevant activities that drive the transition in these domains forward include sustaining a supportive policy environment, creating financial sustainability, developing local stakeholder capacity, communicating to all stakeholders, and aligning programs. Ideally transition monitoring would begin prior to transition processes being implemented and continue for some time after transition has been completed. As no set of indicators will be applicable across all types of health program transitions, we instead propose guiding questions and illustrative quantitative and qualitative indicators to be considered and adapted based on the transition domains identified as most important to the particular health program transition. The M&E of transition faces new and unique challenges, requiring measuring constructs to which evaluators may not be accustomed. Many domains hinge on measuring “intangibles” such as the management of relationships. Monitoring these constructs may require a compromise between rigorous data collection and the involvement of key stakeholders.

**Conclusion:**

Monitoring and evaluating transitions in global health programs can bring conceptual clarity to the transition process, provide a mechanism for accountability, facilitate engagement with local stakeholders, and inform the management of transition through learning. Further investment and stronger methodological work are needed.

## Abstract (precis)

Monitoring and evaluating large-scale global health program transitions can strengthen accountability, facilitate stakeholder engagement, and promote learning about the transition process and how best to manage it. We propose a conceptual framework with 4 main domains relevant to transitions—leadership, financing, programming, and service delivery—along with guiding questions and illustrative indicators to guide users through key aspects of monitoring and evaluating transition. We argue that monitoring and evaluating transitions can bring conceptual clarity to the transition process, provide a mechanism for accountability, facilitate engagement with local stakeholders, and inform the management of transition through learning.

## INTRODUCTION

The donor community has long been interested in the sustainability and fate of public health programs after donor funding is reduced.^1^^,^^2^ This interest has escalated recently, as a result of shifts in donor priorities and the resulting rapid reductions in, and often complete withdrawal of, external funding.

The process of transitioning financing and control of large-scale health programs from donors to local governments is not new. Among programs funded by the United States Government (USG), the transitions of large-scale health programs have been called “graduations” and have been occurring at least since the 1980s with the graduation of family planning assistance programs in Latin America and the Caribbean.^3^^,^^4^ However, transitions are gaining both momentum and interest. For example, reauthorization of the US President’s Emergency Plan for AIDS Relief (PEPFAR) in 2008 reinforced the notion that transition must be handled carefully, with the introduction of Partnership Frameworks, which aimed to ensure PEPFAR programs were sustainable through a renewed focus on “country capacity, ownership and leadership.”^5^^,^^6^ Transitions of PEPFAR programs are ongoing in sites such as South Africa and the Caribbean and are expected to be initiated in other countries in the near future.

USG agencies are not the only ones to engage in such discussions. The Bill and Melinda Gates Foundation, The Global Fund to Fight AIDS, Tuberculosis and Malaria, and Gavi, the Vaccine Alliance, are among several other global organizations with growing interest in the transition from donor assistance toward long-term sustainability. Gavi, for example, has recently revised its graduation policy, which outlines a process for phasing out Gavi support with time-limited catalytic investments to support graduation plans.^7^

The discussion of phasing out donor support often comes after years, if not decades, of investments in strengthening service delivery and health systems and significant efforts to reduce the burden of disease. Poorly executed transitions risk reversing health achievements, negatively affecting services and outcomes for the beneficiary population. As ownership is transitioned from donors to local counterparts, clear accountability is needed to ensure transition is successful. However, with a few exceptions,^8^ transitions to date have been conducted on an ad-hoc basis, where lines of accountability for long-term sustainability between donors and local counterparts have not been clear and systematic, and purposeful monitoring of post-transition health outcomes was not prioritized by the aforementioned stakeholders.^2^^,^^4^^,^^9^^,^^10^

Poorly executed transitions of large-scale global health programs from donors to local governments risk reversing health achievements.

Despite the high stakes involved in transition processes, there are relatively few documented examples of how the transition process was managed, of the effects of transition on the health outcomes of interests, or of monitoring and evaluation (M&E) of how the transition process itself was executed or managed. The US Agency for International Development (USAID) commissioned midterm^11^^,^^12^ and final^10^^,^^13^ evaluations for transition, and compilations of lessons learned,^2^^,^^6^ from selected Latin American and Caribbean countries that graduated from family planning assistance. However, few of these studies are rigorous evaluations, and most could be considered compilations of lessons learned through readily available qualitative and quantitative data sources. Further, most of these evaluations focused specifically on program-related outputs and outcomes. More recently, Gavi has sought to assess the readiness of graduating countries to assume responsibility for sustainable financing of the immunization process.^14^ Attention has also focused on the transitioning of health care worker support from PEPFAR to local funding.^15^ However, the frameworks to define and measure transition processes are few. Recent efforts to develop more systematic approaches to the M&E of transition include an evaluation of the sustainability of Gavi-funded immunization programs in Bosnia-Herzegovina (BiH) after the country became ineligible for Gavi funding,^16^^,^^17^ and efforts to prospectively monitor and evaluate the transition of the Gates-funded Avahan HIV prevention project in India to local ownership.^18^^-^^20^ To date, with the exception of the Avahan transition evaluation, none of the existing M&E activities has examined transition prospectively or throughout the entire transition process,^18^ and there have been no systematic efforts to develop an approach for the M&E of transition.

There have been no systematic efforts to develop an approach to monitor and evaluate transitions of global health programs.

In this paper, we draw on our team’s experiences to develop an initial description of the key dimensions of health program transitions from donors to local counterparts. Furthermore, we make the case for why M&E of the transition process is important and propose a framework to facilitate the identification of different domains and dimensions to monitor and evaluate before, during, and after transition. Finally, we propose guiding questions linked to these domains, which can stimulate thinking around the potential indicators for the M&E of transition. We argue that the M&E of transition can help us obtain a deeper appreciation of how transition unfolds, as well as of the evidence necessary to refine our understanding of how transitions should be planned and managed. We focus on health programs whose goals are to be continued after the transition, unlike, for example, the transition of polio programming after polio eradication.

## METHODS

Our paper takes a reflective “thought exercise” approach similar to that of Gilson et al.,^21^ whereby the content was generated in part through author reflections on professional experiences and refined through discussion with other practitioners. Four authors (SB, DR, SO, LP) have had involvement in monitoring and evaluating the transition of health programs. SB, SO, and DR were involved with monitoring and evaluating the transition of Avahan’s HIV prevention program, and LP was involved in evaluating Gavi support to BiH, whose funding ended prior to the implementation of Gavi’s graduation policy.^18^^-^^20^ This direct involvement in the work allowed for insights on how M&E occurred during the transition process for different sorts of program transitions. In order to help ensure the broader generalizability of our recommendations, we also reviewed peer-reviewed and gray literature on transitions and on the M&E of transition.

Overall, to create the conceptual framework and guidance presented here, we:

- Based our initial thinking on the conceptual framework described in Bennett et al.^18^ for monitoring and evaluating the transition of the Gates-funded Avahan project for HIV prevention.
- Searched the literature including peer-reviewed literature, gray literature, personal author collections, and papers recommended by experts to identify additional relevant sources that would inform the adaptation of the framework and our thinking about the M&E of transition.
- Conducted semi-structured interviews with individuals with experience in health program transitions (N = 6); 5 respondents had firsthand involvement in USAID’s family planning graduation in Latin America, while based either at USG or within implementing partners; 1 respondent was engaged, at the time of the interview, in a CDC-funded activity to measure and monitor the transition of HIV care and treatment from international NGOs to local NGOs, and eventually to the country government.
- Adapted the Avahan framework^18^ iteratively to develop a revised version informed by the literature, semi-structured interviews, and authors’ general experience with M&E of transitions, and developed guidance on approaching the M&E of transition.
- Obtained two rounds of feedback (27 June 2014, 12 September 2014) about the draft framework and guidance from a group of key informants (N = 5, separate from the interviewees noted earlier) with experience in health program transition; these informants were selected based on their recent engagement in the development of the Gavi graduation policy (n = 1), their knowledge of the scaling down of PEPFAR activities in the Caribbean (n = 2) and in Namibia (n = 1), and their knowledge of M&E and possible sources of indicators that could be used to monitor or evaluate transition processes (n = 1); all informants were associated with implementing, rather than funding, agencies. During both meetings, feedback was sought on how to package the available evidence around M&E of transition, the components of the proposed framework, and possible indicators and sources of indicators for each of the framework domains.
- Finalized the conceptual framework and guiding questions for this paper.

Ultimately, the final framework and approach presented here is meant to build on previous experiences in implementing and managing M&E of transitions, so as to present a guide for how those engaged in transition can think about the process, as well as for program evaluators and planners on possible approaches to the M&E of future large-scale health program transitions.

## FINDINGS

Our findings are organized as follows. First, we present a conceptual framework highlighting potential transition domains, activities, and outcomes to be monitored or evaluated. Next, based on this framework, we describe possible approaches to the M&E of transition including reflections on timing and organization of M&E approaches. Finally, we suggest guiding questions and potential indicators that might be used to monitor and evaluate health program transition.

### Conceptual Framework

The proposed conceptual framework (Figure) seeks to guide users through key aspects of monitoring and evaluating transition in a comprehensive fashion. It aims to be broad and inclusive of elements relevant to a large-scale health program transition, although we recognize that transitions take many forms and that the nature of transition drives which domains become pertinent for each particular case.

**FIGURE:**
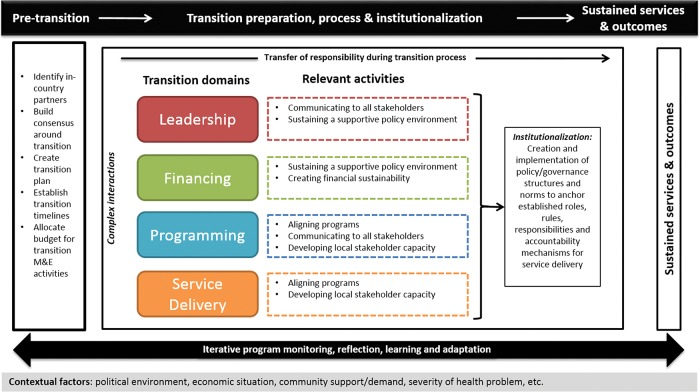
Conceptual Framework for Large-Scale Global Health Program Transitions

We conceive of transition as a process. Pre-transition activities set the parameters for transition including key factors such as the timeline, budget, and partners involved. Subsequent to these pre-transition activities, the transition entails a set of ongoing iterative processes that gradually shift program responsibility from the donor to the program recipient. Transitions may occur across one or more domains: leadership, financing, programming, and service delivery. Each of these domains is explained more fully in Table 1.

Transitions can occur across one or more of 4 domains: leadership, financing, programming, and service delivery.

**TABLE 1:** Transition Domains Explained

Transition Domain	Explanation
Leadership	High-level leaders must accept that the transition process is actually occurring, and health sector leaders need to provide political support for transition and for sustaining the program in its new environment post-transition. Political will and commitment are complex and context-specific and need to be built beyond individual leaders, who may be transient. High-level leadership needs to come from both within the health sector and from non-health sector actors, such as the Ministry of Finance.
Financing	In order to ensure financial sustainability post-transition, the program recipient will need to identify and secure new sources of funding as prior sources of financial support are eliminated. Funding will likely come from multiple sources, and, as such, activities may include lobbying to secure funding from new sources and creating and altering financial mechanisms for improved sustainability.
Programming	Responsibilities for program management, such as day-to-day operations, as well as staff management, funding, reporting requirements, monitoring and evaluation, and other administrative tasks, must be transitioned, to the extent that such functions were previously provided by donors. Capacity assessments can help diagnose competencies and signal the amount of capacity building and training required to transition programming.
Service delivery	In instances where donors, and not local organizations, have been directly responsible for service delivery, the local program recipient may have to take responsibility for the logistics of service delivery, including human resources, commodity procurement, community outreach, and other elements related to the program services itself.

For each transition domain, we identified a set of relevant activities, which help to anchor the necessary responsibilities, rules, norms, and structures into the program recipient environment. Ultimately, these activities facilitate the delivery of program services by the recipient, at a level defined by the transition goals. These activities include:

**Sustaining a supportive policy environment:** Ideally transition plans are developed and executed in an environment where existing policies and involved stakeholders are committed and supportive of achieving the overall objective of the transition and of the health program. In practice, transitions can be politically motivated and implemented abruptly and are often met with resistance and/or disbelief. Therefore, it is crucial for transition planning to conduct activities strategically to build political commitment and support.^9^ In instances where leaders can be held accountable to their constituents, creating wide public support for a health program may encourage leaders to visibly support the sustainability and transition of a health program. In other instances, “soft” approaches such as strategic communication of benefits could be applied to influence powerful stakeholders. Signals of a supportive policy environment may include the post-transition program being embedded in national policy or specific program goals being reflected in national and/or subnational plans and budgets. Sometimes existing policies may undermine program sustainability; for example, existing policies inhibiting effective procurement processes would be a target for change.

**Creating financial sustainability:** The existence of secure and diversified funding is central to the sustainability of a health program.^22^^,^^23^ The burden of securing this funding is dependent on the context, but the responsibility may fall in part on the program recipients themselves as well as on donor agencies, and potentially on program beneficiaries. As there can be multiple donors and funding sources for a program, coordination is central in organizing how funds are raised and shared. An understanding of a country and program’s current and future needs (e.g., using a resource plan) can better conceptualize the funding situation to key audiences.^24^ Ultimately, funding is related to contextual issues (economic conditions, political will, competing political/government priorities, capacity) and is affected by the donor landscape, where the presence of generous donors may discourage the acceptance of financial responsibility.^25^

**Developing local stakeholder capacity:** Shifting health program responsibility from donor to program recipient means that the capacity previously supplied by donors must be replaced or adapted according to the priorities and capacities of local actors. Ideally, developing local capacity and building ownership is a process initiated long before transition begins; however, in practice, this is not always the case. Organizational capacity assessments may diagnose existing competencies and identify areas in need of investment to reach sufficient capacity for sustaining the health program.^26^ When capacity is insufficient, capacity-building activities should be initiated to develop the necessary components to continually deliver program activities.^26^ Capacity goes beyond the idea of having the skills and tools to deliver program activities to also comprising staff, facilities, structures, and systems. A diverse range of activities to support this domain can be undertaken, depending on the needs and resources present.^27^

Developing local capacity to ensure effective transitions goes beyond strengthening the skills and tools to deliver program activities.

**Communicating to all stakeholders:** As the transition process inherently involves shifts in power and authority, it often faces considerable stakeholder resistance. Timely, transparent, and appropriately disseminated communication plays a key role in persuading stakeholders and forming a group of proponents to support transition. Communication helps to align expectations, forge common goals, and facilitate building positive relationships among key audiences. These activities can help to overcome common risks of transition, such as being overwhelmed by tension and confusion from misinformed audiences, as well as an overall resistance to transition.^28^ Communication of the transition plan needs to occur at multiple levels, from the donor to senior management, as well as from program recipient to frontline workers. Poor communication with frontline workers can create resistance due to shifting priorities or changing values.

**Aligning programs:** Programs may need to undergo a process of harmonization with existing services as they transition. This harmonization can include the adaptation of program services as well as the implementation of common arrangements for planning, management, financial reporting, and M&E, so as to integrate with the national program or host environment. Programs activities can be adapted, completely removed, or remain unchanged in accordance with the program recipient and health system context.^29^ The implementation of such alignment processes can constitute a significant task.

The 5 activity areas above are closely interconnected and reflect complex adaptive relationships that influence one another.^30^ For example, communicating transition plans to stakeholders is closely related to creating and sustaining a supportive policy environment, while the policy environment influences a program’s ability to secure funding and align programs with existing ones.

Together, these activities drive the transition of leadership, financing, programming, and service delivery forward so that program recipients can take full responsibility over these domains and over the health program as a whole. The intermediate result is the institutionalization of the program, by which we mean the development of policies, norms, and structures to sustain the program within the recipient organization’s health system. In total, a well-managed transition process enables the sustained delivery of program services and, hence, sustained health outcomes, even if there may be changes in who delivers services or the mechanisms through which they are provided. Ideally, a transition plan including a context-specific set of activities would be developed jointly in advance of transition and agreed upon between donors and recipient countries. Such a plan would clearly facilitate the M&E of transition, but historically such a document has been rare.

### Approaches to Monitoring and Evaluating Transition

The conceptual framework described in this article can be used to:

- Identify the most relevant domains being transitioned
- Assess which activities might be prioritized for monitoring
- Formulate appropriate indicators for monitoring and evaluating transition using the proposed guiding questions explained below

The framework may also help program planners and evaluators to reflect on the question of when in the transition cycle it will be important to have measures of transition. As for many evaluations, determining the purpose of M&E is likely to be a critical first step.^31^ For example, if the M&E process is meant to help make course amendments to transition plans, then measures of readiness for transition (perhaps reflecting the extent to which transition activities have been implemented as planned, and how they have affected the 4 transition domains) may be key. Alternatively, if the purpose of M&E is to help hold key stakeholders, such as donors and local counterparts, accountable for what happens post-transition, then the focus of M&E may be on measures of institutionalization and service outcomes. For M&E that is designed to aid learning processes and cast light on what constitutes effective transition practice, it will likely be important to have measures of both transition activities and institutionalization and outcomes to allow investigation of how transition activities affect final outcomes. Regardless of the purpose of M&E, it will be important for program planners and evaluators to facilitate clear opportunities for stakeholder engagement (i.e., both donors and recipients), before, during, and after the transition.

Not all health program transitions will reflect all of the domains described in the conceptual framework. Table 2 describes the relevant domains for 3 different health program transitions with which we are familiar (Gavi graduation, USAID family planning transitions in Latin America, and the Avahan transition). The table illustrates that for some health program transitions (such as Avahan), all 4 of the transition domains will be relevant, whereas for others, such as Gavi, the focus may be on a more limited number of domains.

**TABLE 2:** Transition Types and Implications for Monitoring and Evaluation

	Transition Type		
	Gavi in Bosnia-Herzegovina	FP in Latin America	Avahan in India
**Transition Description**	From 2002 to 2011, Gavi supported the government of BiH to introduce the Hepatitis B and Hib vaccines, which were delivered through the government health system. BiH passed Gavi’s GNI per capita threshold in 2007, making it ineligible for new support, while Gavi fulfilled existing multiyear commitments. The government of BiH assumed funding and planning responsibilities from Gavi when the funding ended, which was before Gavi developed a graduation policy.	Through USAID, the USG supported FP activities in LAC through financial and technical assistance beginning in the 1960s. In the mid-2000s, due to shifting donor priorities and improving FP indicators, countries were systematically “graduated” from FP assistance. Transition plans, typically spanning 2–5 years, were developed where funding and procurement was transitioned to local in-country organizations.^2^	In 2005, the BMGF committed US$350 million to address the spread of HIV/AIDS in India, focusing on prevention for high-risk populations. The programs, in 6 states, offered services through cascading contracts with international and local NGOs. A planned and phased program transition took place between 2009 and 2012.
**Transition Domain**			
Leadership	X	X	X
Financing	X	X	X
Programming		X	X
Service delivery			X
**Key M&E Dimensions**	**⟶ INCREASING COMPLEXITY ⟶**		
Sustaining a supportive policy environment	X	X	X
Creating financial sustainability	X	X	X
Developing local capacity	X	X	X
Communicating among all stakeholders		X	X
Aligning programs			X

Abbreviations: BiH, Bosnia-Herzegovina; BMGF, Bill and Melinda Gates Foundation; FP, family planning; GNI, gross national income; Hib, *Haemophilus influenzae* type B; LAC, Latin America and the Caribbean; M&E, monitoring and evaluation; USAID, US Agency for International Development; USG, United States Government.

In an ideal situation, the monitoring of transition would begin prior to transition processes being implemented and continue for some time after transition has been completed. The pre-transition period would be used to engage key stakeholders and reach consensus on plans for transition and M&E of transition. This period may also be used for reflection on what type of transition will be taking place and over what timeline. During this period, donors and potential program recipients should engage in open and transparent discussions to develop consensus around the program’s transition goals and M&E plans.^32^ This process should determine transition stakeholders, review the reasons for why transition is occurring, allocate a budget for transition activities, and ultimately create an agreed-upon transition and M&E plan. Based on transition experiences to date, it is not possible to recommend a more specific time frame for when M&E of transition should begin. Based on the authors’ experience, Avahan began its preparations for transition 2 years prior to the first wave of transition and 5 years prior to the main transition round. Gavi is currently looking to conduct transition assessments in countries as soon as their gross national income (GNI) per capita rises above the low-income country threshold, which, according to our observations, occurs roughly 5 years before graduation. The post-transition period is equally important and should be used to monitor sustainability of outcomes and identify potential unintended consequences. Our review of transition experiences has allowed us to reflect on key principles to be considered when monitoring and evaluating the transition process (Box).

The monitoring of transition should ideally begin prior to the implementation of transition and should continue beyond the completion of transition.

**BOX. Principles for Monitoring and Evaluating Health Program Transitions**

- **Establish clear end goals.** Clear end goals are critical to guiding M&E plans. Stakeholders should clarify their vision for the future of the program and of its end goals with respect to service coverage and health outcomes.
- **Plan early.** Early planning allows for regular and consistent monitoring of the transition process, evaluation of transition preparation activities, and collection of baseline data to determine impacts post-transition.
- **Ensure program recipients are vested in M&E.** It is critical to engage all stakeholders and secure commitments from key actors regarding the use of M&E evidence. It is especially important for the program recipient to engage in the process to ensure access to data post-transition and also given their central role in acting upon M&E evidence.
- **Earmark funding for transition M&E.** Programmatic transition is distinct from programmatic service delivery and, as such, needs specific earmarked funding, as does the M&E of transition.
- **Protect the neutrality and independence of evaluators.** To promote acceptance of, and action on, M&E findings, all stakeholders need to view M&E results as unbiased and independent. External evaluators may help achieve this perception, but evaluation teams composed of donor and program recipient representatives may also be appropriate.

Often the triggers for transition will influence the nature of the entire process and the time frame within which it is implemented. For example, donors may signal the need for transition on the basis of target indicators being met, or for political reasons. In other cases, program recipients may initiate transition planning as part of developing a sustainable program. Longer time frames facilitate better planning for transition and stronger transition M&E that, in turn, allow for deeper learning. However, not all transitions will take place under ideal circumstances, and sometimes they may be hurried, responding to political imperatives rather than to carefully determined and mutually agreed conditions. While all transition circumstances offer opportunities for monitoring and for learning, rapid transitions typically limit the scope for rigorous evaluation and may be associated with antipathy toward learning. The costs associated with the M&E of transition will vary according to the scope and scale of the exercise, varying from modest, highly focused efforts that address just one phase of the transition cycle (such as transition preparedness) through to more comprehensive M&E processes, spanning into the post-transition period.

## Guiding Questions and Indicators

No set of indicators will be applicable across all types of health program transitions. Accordingly, rather than proposing a short list of indicators, we present guiding questions and selected illustrative indicators to be considered and adapted. We encourage the use of both quantitative indicators and qualitative investigation as complementary approaches necessary to fully explore transition. Quantitative indicators identify changes that have occurred due to transition, demonstrate trends, and track whether transition goals, objectives, and milestones are being met. Qualitative methods describe transition experiences, explain why changes have occurred and their repercussions, and indicate what feedback and adaptation are taking place. Such a qualitative investigation can be critical to understanding why unexpected effects are occurring or to identifying the underlying causes of poor transition performance.

Both quantitative and qualitative data are needed to fully understand the transition process and its effect on the wider health system.

The selection of indicators for monitoring should be driven primarily by the importance of what is being measured; the scientific soundness of the measure; and the feasibility of obtaining data on the measure.^25^ In terms of importance, ideally the transition planning process will have developed a clear logic model (perhaps building on the conceptual framework presented here) that describes the anticipated linkages between transition preparation activities and outcomes. In such a context, it will be rational to tie the selection of indicators to the main constructs covered in this context-specific logic model, ensuring a balanced set of indicators across the different aspects of the transition identified as important. For example, using our conceptual framework, monitoring indicators could seek to capture a variety of pre-transition activities (such as development of a transition plan); aspects of the transition preparation process (such as measures of local stakeholder capacity, program alignment, communication, etc.); the extent of program institutionalization; and measures of outcomes (both service coverage and health outcomes) and how they are sustained over time. In situations where there is not a clearly defined transition plan, those planning the M&E of transition will need to piece together the pre-transition activities that would be underway to prepare for transition.

Health program evaluators are likely accustomed to measuring indicators related to health services and outcomes, and such data are often routinely collected as part of program M&E processes. However, the M&E of transition requires understanding of quantitative indicators that may lie beyond typical health program indicators and are likely to be scattered among different stakeholders and data sources. Table 3 lists possible quantitative indicators of transition M&E.^18^^,^^33^^-^^36^

**TABLE 3:** Guiding Questions for Monitoring and Evaluating Global Health Program Transitions and Illustrative Indicators by Transition Domain

Domain	Guiding Questions	Sample Indicators (obtained through quantitative and qualitative inquiry)
**CONTEXTUAL FACTORS**	To what extent is the political environment ready for a health program transition?	Score on World Bank Governance Index % government budget spent on health % government budget spent on health program of interest
	To what extent is the economic situation ready for a health program transition?	GNI per capita USD per capita spent on health
	To what extent is there community support for the health program to transition?	Civil society engagement in health program
	To what extent is the severity and scope of the health problem addressed by the program to transition?	% geographic coverage of program # deaths or cases averted due to health program % service delivery coverage target addressed by health program # vulnerable populations reached by health program
**PRE-TRANSITION**	To what extent has a core set of transition stakeholders been identified?	Donor and program recipient have agreed on key stakeholders for transition, including communities/beneficiaries, civil society, etc. Transition team representing key stakeholders has been established
	To what extent has this core set of transition stakeholders agreed on transition objectives?	% key stakeholders who have participated in transition planning events
	To what extent have the transition objectives been planned for, including monitoring and evaluation?	Transition plan with M&E has been agreed upon and documented, including transition timelines
	To what extent have budget allocations been made for transition, including M&E of transition?	% program recipient transition budget that has been funded M&E transition budget available
**TRANSITION**		
**Leadership**	To what extent is there clear commitment from the political level for program service delivery over the long term?	Program is integrated into national policy or health plans % leaders of affected communities who have been informed of transition plans
	To what extent is there transparent government leadership and management?	Guidelines allow exceptions to operating norms based on realities on the ground Clear lines of government accountability exist for the health program
	To what extent have local authorities incorporated the demands of program service delivery into their routine operations?	% program activities integrated into local operational plans
	To what extent do local stakeholders believe that the health program is a valuable and effective investment of their time and resources?	% program implementers who believe that program recipient has the same or higher level of commitment toward the program as the donor
**Financing**	To what extent does the program recipient have transparent systems to develop and maintain budgets and expenditures?	% implementers with an audit of their financial records
	To what extent have financial responsibilities been transferred from donor to program recipient?	% donor contribution to health program versus government funding Any recent or planned transitions from other donors working in health area
	To what extent has program recipient secured adequate funding to sustain program?	% gap between estimated annual program costs and resources available
**Programming**	To what extent is there technical, managerial, and financial capacity within the program recipient to effectively deliver key health program services?	% of required supervision sessions that occur % health program recipient staff qualified for financial management % supervisory or managerial position vacant at health program recipient
	To what extent have any shortages in capacity been identified?	Capacity needs assessment of program recipient has been conducted
	To what extent are training/capacity-building activities occurring or planned to address gaps in capacity?	% training activities completed where capacity shortages were identified
**Service delivery**	To what extent are budgetary and financial systems aligned with those of the program recipients?	Overall budget and individual line items are reviewed and adjusted for alignment
	To what extent are reporting structures aligned with those of the program recipients?	Reporting frequency of government and program recipients are aligned % reports that are complete upon submission
	To what extent are service delivery or procurement guidelines aligned with those of the program recipients?	% health facilities employing government procurement guidelines
	To what extent do the program M&E systems align with the host country’s M&E systems?	% donor indicators currently being reported to government health monitoring information system
**INSTITUTIONALIZATION**	To what extent are the key features of the original service maintained in the program post-transition?	% key features of the health program that continue post-transition
	Is there a regular budget line and allocation to support implementation of this program?	Budgets at national/district/facility level reflect funding necessary to support transitioned program Budgeted funds are allocated and disbursed in a timely fashion.
	To what extent is the program reflected in routine norms and guidelines?	Government standard operating procedures reflect modalities of the transitioned program
	Is the health program viewed as a success by key administrators and program implementers?	% key program administrators and implementers who view the program as a success
**SUSTAINED SERVICES AND OUTCOMES**	To what extent is the program recipient controlling and managing delivery of essential program services?	% health program services delivered through program recipient facilities
	How has the quality of program services changed?	% clients who are satisfied with the program’s services % program administrators and implementers describing same or improved quality of program services post-transition
	How has the coverage of program services changed after transition?	# health facilities providing service before, during, and after the transition
	How have key outcome indicators and key health outcome indicators relating to the health program changed?	Prevalence and incidence of health condition in question Coverage of vulnerable populations reached by the health program
	How was the transition experience overall?	% program administrators and implementers who suggest the overall program changed significantly as compared with pre-transition % program administrators and implementers describing the transition as smooth

Many of the guiding questions for quantitative indicators could also be explored through qualitative methods. We have included in Table 3 illustrative questions that can be used in semi-structured interviews or focus group discussions with key stakeholders who are either engaged in or affected by transition.

## DISCUSSION

Based on our collective experiences of transition, including interviews and key informant discussions combined with a literature review, we developed a conceptual framework that broadly details how the transition process for large-scale health programs occurs and offers an approach to identifying what, beyond traditional M&E impact indicators, can be monitored throughout the transition process. The 4 domains of transition of leadership, financing, programming, and service delivery involve sets of activities to be considered during a transition process—from sustaining a supportive policy environment and creating financial sustainability to developing local stakeholder capacity, communicating the transition plan to all stakeholders, and aligning programs—which will require active and evidence-informed management. By monitoring and evaluating these activities during transition and using the resulting evidence for decision making, planners may gain a better sense of where attention needs to be focused or shifted during the transition process, making way for any necessary course correction.

While the ultimate goal of transition, namely, sustaining or enhancing services and outcomes, reflects constructs that health program evaluators are accustomed to measuring, other dimensions of transition may present challenges for evaluators. M&E of transition requires consideration of measures of factors that are much less commonly assessed in health program evaluations such as program alignment, the presence of a supportive policy environment, organizational capacity, and effective communication. Further, likely measures of these factors may be embedded in various data sources that are scattered across different stakeholders and often cannot easily be identified through routine M&E channels. Indeed, M&E of transition may require the development and implementation of special data collection tools.

M&E of transition requires measurement of factors that are not as commonly assessed in traditional M&E of health programs.

Health programs typically emphasize rigorous and scientific M&E methodologies to ensure high data quality and accuracy. However, transition processes may differ in the sense that they rely heavily on the effective management of relationships between different stakeholders, most notably between donors and the recipient organization. Thus, when designing an approach for M&E, it may be beneficial and necessary to allow for a trade-off between scientifically rigorous data collection and quality metrics on the one hand and involvement of the right stakeholders on the other hand. Poor data collection processes or quality can gradually be improved, but there is no substitute for involving the right stakeholders from the outset.

The transition process is rarely easy as organizational change is occurring at various levels in a health program. Thus, introducing M&E as an additional component in the process of transition holds several challenges unique to the transition context. First, a lack of buy-in from program recipients can hinder M&E of transition as evaluators may be unable to access post-transition data to assess effects on service delivery and impact after transition. Second, breakdowns in the relationship between donor and program recipients may hinder the accessibility of those conducting M&E as the program is transitioning to the program recipient. Further, and similar to regular evaluations, it will be critical to have reliable baseline data for transition M&E, where it may be particularly important not to assume that performance of the program prior to transition was optimal. Third, although the resources for managing the transition process itself may be easier to obtain, dedicated financial and human resources for transition M&E may be more difficult to secure during a time of financial constraints. M&E of the post-transition period would be most difficult to manage. Donors might have limited resources and leverage to request support for and engagement in the M&E of the post-transition period, given the withdrawal of funding. Arrangements for access to M&E data post-transition should be ideally negotiated and agreed to during the pre-transition period, while the donor maintains influence on programming. Fourth, different dimensions that are monitored during transition change at different speeds. Dimensions such as political commitment can change rapidly while others such as local stakeholder capacity change more predictably. Those involved in M&E need to consider what timelines are practical for data collection and analysis, as well as how often recommendations and feedback should be provided to stakeholders. Finally, there may be resistance to transition from within the donor organization, its implementing partners, and/or the program recipient organization. Transition shifts power and resources, creates additional work, and may bring about unwelcome shifts in organizational priorities. All of these factors may create resentment and negativity toward the transition process, and, by extension, to the M&E of transition.

### Limitations

Our study faces several limitations. Our paper is based in part on author experience, creating the potential for biased interpretations and presentations. However, given the limited literature on the M&E of transition, we felt that integrating stakeholder interviews and consultations with our own experience in the field was critical in presenting a balanced final product. Also, although our framework is based on collective practical experiences, it has not been used prospectively in any program transition. Without having piloted the framework in the field, we are uncertain of the final utility of our thinking, and thus, we seek feedback on experience with applying this framework to monitor and evaluate health program transition. Finally, in this paper we focused our discussion on the transition of large-scale programs (e.g., family planning, immunizations) and did not explore the transition of small-scale projects.

## CONCLUSIONS

Transition M&E can offer important benefits. The discipline of thinking through what transition entails and how best to describe and measure it can provide greater conceptual clarity to the whole transition process. M&E of transition can also help inform countries undergoing transition about how best to manage it—in terms of learning not only from other countries that have undergone the process but also from their own transitions over time. Such real-time learning through M&E can help identify potential problem areas before they manifest into more serious issues. M&E of transition may also provide an element of accountability for donors, allowing them to be assured that the transition process was executed with attention to detail and with overall sustainability of the program in mind. Finally, M&E of transition allows an opportunity to engage extensively with local stakeholders in the process of transition, ensuring that concerns and needs are appropriately shared as the program is being transitioned to local ownership.

Given the major shifts currently taking place in the development assistance landscape, ensuring effective health program transitions that sustain key health outcomes is likely to be a high priority for years to come. To date, M&E of transition processes has been relatively neglected, thus constraining the ability to learn from transition. Greater investments and stronger methodological work on the M&E of health program transition are needed. Piloting the proposed framework and other approaches for M&E transition would be one of the important first steps assisting our collective thinking about how to ensure that the accomplishments and health gains to date are not compromised during upcoming transitions.

Greater investments and strong methodological work on M&E of transition are needed.

## Notes

**Peer Reviewed**
